# Association of Psoriasis With Thyroid Disorders: A Hospital-Based, Cross-Sectional Study

**DOI:** 10.7759/cureus.22987

**Published:** 2022-03-09

**Authors:** Deepak Yumnam, Naveen Kumar Kansal, Ravi Kant

**Affiliations:** 1 Dermatology, All India Institute of Medical Sciences, Rishikesh, Rishikesh, IND; 2 General Medicine and College of Nursing, All India Institute of Medical Sciences, Rishikesh, Rishikesh, IND

**Keywords:** thyroid disorders, thyroid autoimmunity, hypothyroidism, hyperthyroidism, psoriasis

## Abstract

Background

Although numerous studies have been conducted to determine the relationship between psoriasis and thyroid dysfunction, contrasting results have been reported. The link between psoriasis and thyroid disease has not been elucidated well. This study aimed to determine the frequency of thyroid dysfunction and its relationship with gender, age, duration, and severity of psoriasis among patients with psoriasis.

Methodology

Psoriasis was diagnosed clinically, and the severity of the disease was assessed by the Psoriasis Area Severity Index (PASI) score and the body surface area (BSA) involved, as measured by Wallace’s rule of nine. A total of 111 patients with psoriasis were analyzed for thyroid dysfunction, which included thyroid-stimulating hormone, free T3, free T4, and anti-thyroid peroxidase (anti-TPO) antibody levels. SPSS version 23.0 (IBM Corp., Armonk, NY, USA) was used for analysis.

Results

Out of the 111 analyzed psoriasis patients, deranged thyroid status was observed in 22 patients. Anti-TPO was increased in 19 patients. Patients with thyroid dysfunction had a more severe form of psoriasis (both by PASI score and BSA involvement) than those with mild psoriasis (61.9% vs. 38.1%), whereas patients with increased anti-TPO had a milder disease.

Conclusions

This study illustrated deranged thyroid status and thyroid autoimmunity in 19.8% and 17.1% of psoriasis patients, respectively, suggesting thyroid dysfunction and autoimmunity. However, further studies are required to expand our knowledge of psoriasis and its association with thyroid dysfunction and thyroid autoimmunity, as well as with age, gender, disease duration, and severity of psoriasis.

## Introduction

Psoriasis is a chronic, immunologically mediated, proliferative skin disorder. Various studies have revealed that autoimmune diseases are more prevalent in patients with psoriasis and psoriatic arthritis than in the general population. Individuals with psoriasis are almost twice as likely to develop another autoimmune condition than those without psoriasis [1]. Autoimmune disorders linked with psoriasis include vitiligo, diabetes mellitus, autoimmune thyroiditis, rheumatoid arthritis, and inflammatory bowel disease. Patients with psoriasis have an increased incidence of thyroid diseases, including hyperthyroidism, hypothyroidism, Graves’ disease, and Hashimoto thyroiditis [1]. Graves’ and Hashimoto’s disease are the most common cause of hyperthyroidism and hypothyroidism, respectively, in iodine-replete areas. Among thyroid antibodies, anti-thyroid peroxidase (anti-TPO) antibodies are the most frequent autoantibodies in patients with autoimmune thyroid disease (AITD), present in approximately 90% of the patients with Hashimoto’s thyroiditis. Although the exact link in the pathogenesis is not well understood, both AITD and psoriasis share several similar inflammatory pathways, which is possibly the connection between the two disorders. Genetic, environmental, immune defects, and hormonal factors participate in the pathogenesis of psoriasis. It is postulated that thyroid hormones bind to the receptors present on the skin and produce epidermal growth factor (EGF), which leads to keratin synthesis and epidermal hyperplasia [2,3]. Several studies have implicated the role of T-helper type 1 (TH-1) cell-mediated inflammation and their various inflammatory pathways in the immunopathogenesis of psoriasis and AITD [4]. There is no current evidence regarding the association of human leukocyte antigen (HLA). Our study intends to assess the frequency of thyroid dysfunction and thyroid autoimmunity in patients with psoriasis and determine the association among thyroid dysfunction, thyroid autoimmunity, and clinical features, including gender, age, duration, and severity of psoriasis.

## Materials and methods

We included patients with psoriasis of any clinical type who presented consecutively to the Dermatology Department from 2020 to 2021 and consented to participate in this study. Pregnant or lactating mothers were excluded from the study. The study was approved by the Institutional Ethics Committee (Registration number: 337/IEC/PGM/2020). Clinicodemographic profile, history, and disease severity were recorded. Psoriasis was diagnosed clinically, and the disease severity was assessed by the Psoriasis Area Severity Index (PASI) score and body surface area (BSA) involved, as measured by Wallace’s rule of nine at the time of initial presentation. PASI score of >12 and BSA involvement of >10 were defined as severe. Blood samples were collected on an empty stomach. Thyroid-stimulating hormone (TSH), free T3 (FT3), free T4 (FT4), and anti-TPO were measured using Advia Centaur XP immunoassay (Siemens, Munich, Germany). TSH of 0.35-5.5 µlU/mL, FT3 of 2.3-4.2 pg/mL, FT4 of 0.89-1.76 ng/mL, and anti-TPO of 0-60 U/mL were taken as the normal reference range. Anti-thyroglobulin antibodies and anti-thyroid stimulating hormone receptor antibodies were not measured as they were not available in our Institute. Subclinical hypothyroidism was defined as high TSH and normal FT3 and FT4 levels, subclinical hyperthyroidism as low TSH and normal FT3 and FT4 levels, hypothyroidism as high TSH and low FT3 and FT4 levels, and hyperthyroidism as low TSH and high FT3 and FT4 levels.

Statistical analysis

SPSS version 23.0 (IBM Corp., Armonk, NY, USA) was used for analysis. Qualitative variables were summarized as number (N) and frequencies (%), while quantitative variables were summarized as mean and standard deviation (M ± SD). Wilcoxon-Mann-Whitney U test, chi-squared test, and Fisher’s exact test were used for correlating the variables. A p-value of <0.05 calculated at 5% level (95% confidence interval) was considered significant.

## Results

Table 1 depicts the baseline clinicodemographic features and thyroid function abnormalities of 111 psoriasis patients. Of the 111 analyzed patients, 86 (77.5%) were males and 25 (22.5%) were females. The mean age (years) was 39.86 ± 13.84, ranging from 18 to 71 years. In total, 61 (55.0%) patients were aged ≤40 years (type 1 psoriasis) and 50 (45.0%) were aged >40 years (type 2 psoriasis). The mean total duration of illness (years) was 5.35 ± 5.49, with the majority presenting within five years. In most patients, the scalp was the initial site of involvement (47.7%). Overall, 98 (88.3.5%) patients had plaque-type psoriasis, followed by palmoplantar psoriasis (6.3%), erythrodermic (1.8%), guttate (1.8%), and pustular psoriasis (1.8%). In total, 29 (26.1%) patients experienced winter exacerbation, and 13 (11.7.4%) experienced summer exacerbation. The mean PASI score was 10.57 ± 9.61. Overall, 74 (67.9%) patients had a PASI score of ≤12, and 35 (32.1%) had a PASI score of >12 (severe). The mean BSA (%) was 11.95 ± 15.16. Overall, 73 (65.8%) patients had BSA of ≤10% and 38 (34.2%) had BSA of >10% (severe).

**Table 1:** A summary of clinical details. PASI: Psoriasis Area and Severity Index; BSA: body surface area

Clinical parameters	frequency
Age (years) (39.86 ± 13.84)	
≤40 years	61 (55.0%)
>40 years	50 (45.0%)
Gender	
Male	86 (77.5%)
Female	25 (22.5%)
Total duration of illness (years)	
≤5 years	78 (70.3%)
>5 years	33 (29.7%)
Initial site of involvement	
Scalp	53 (47.7%)
Lower limbs	29 (26.1%)
Upper limbs	18 (16.2%)
Trunk	11 (9.9%)
Type of psoriasis	
Plaque psoriasis	98 (88.3%)
Palmoplantar	7 (6.3%)
Erythrodermic	2 (1.8%)
Guttate	2 (1.8%)
Pustular psoriasis	2 (1.8%)
Seasonal exacerbation	
None	64 (57.7%)
Winter	29 (26.1%)
Summer	13 (11.7%)
Monsoon	5 (4.5%)
PASI score (10.57 ± 9.61)	
≤12	74 (67.9%)
>12	35 (32.1%)
BSA (%) (11.95 ± 15.16)	
≤10%	73 (65.8%)
>10%	38 (34.2%)

Table 2 shows the summary of the thyroid function test. In total, six (5.4%) patients had low TSH, and 16 (14.4%) patients had high TSH. Overall, 12 (10.8%) patients had low FT3, and two (1.8%) patients had high FT3. Seven (6.3%) patients had low FT4, and four (3.6%) had high FT4. Anti-TPO increased in 19 (17.1%) patients. Out of the 111 psoriasis patients, 22 (19.8%) patients had deranged thyroid functions; seven (6.3%) subclinical hypothyroidism, eight (7.2%) hypothyroidism, five (4.5%) subclinical hyperthyroidism, and two (1.8%) hyperthyroidism.

**Table 2:** Summary of thyroid function tests. TFT: thyroid function test; TSH: thyroid-stimulating hormone; WNL: within normal limit; FT3: free T3; FT4: free T4; anti-TPO: anti-thyroid peroxidase

Thyroid function tests	Frequency
TSH	
Low	6 (5.4%)
WNL	89 (80.2%)
High	16 (14.4%)
FT3	
Low	12 (10.8%)
WNL	97 (87.4%)
High	2 (1.8%)
FT4	
Low	7 (6.3%)
WNL	100 (90.1%)
High	4 (3.6%)
Anti-TPO	
WNL	92 (82.9%)
High	19 (17.1%)
TFT	
WNL	89 (80.2%)
Subclinical hypothyroidism	7 (6.3%)
Hypothyroidism	8 (7.2%)
Subclinical hyperthyroidism	5 (4.5%)
Hyperthyroidism	2 (1.8%)
TFT impression	
Normal	89 (80.2%)
Abnormal	22 (19.8%)

Table 3 shows the comparative clinicoepidemiologic features of patients with normal thyroid function tests and thyroid dysfunction. A more significant number of males than females (81.8% vs.18.2%), patients aged 40 years or below than older patients (63.6% vs. 36.4%), having psoriasis for ≤5 years than more than five years (72.7% vs. 27.3%), and moderate-to-severe psoriasis (both by PASI score and BSA involvement) than mild psoriasis (61.9% vs. 38.1%) showed thyroid dysfunction. However, there was no statistically significant difference in gender, age, duration, and severity of psoriasis compared to patients with normal thyroid function tests.

**Table 3:** Association between thyroid function tests and clinical parameters. ^1^Wilcoxon-Mann-Whitney U test; ^2^Chi-squared test; ^3^Fisher’s exact test TFT: thyroid function test; PASI: Psoriasis Area and Severity Index; BSA: body surface area; TSH: thyroid-stimulating hormone; WNL: within normal limit; FT3: free T3; FT4: free T4; anti-TPO: anti-thyroid peroxidase

Parameters	TFT impression		P-value
	Normal (n = 89)	Abnormal (n = 22)	
Age (years)	40.44 ± 14.22	37.55 ± 12.19	0.424^1^
Age			0.361^2^
≤40 years	47 (52.8%)	14 (63.6%)	
>40 years	42 (47.2%)	8 (36.4%)	
Gender			0.777^3^
Male	68 (76.4%)	18 (81.8%)	
Female	21 (23.6%)	4 (18.2%)	
Total duration of illness (years)	5.47 ± 5.88	4.86 ± 3.63	0.593^1^
Total duration of illness			0.778^2^
≤5 years	62 (69.7%)	16 (72.7%)	
>5 years	27 (30.3%)	6 (27.3%)	
Type of psoriasis			0.186^3^
Plaque psoriasis	80 (89.9%)	18 (81.8%)	
Palmoplantar	6 (6.7%)	1 (4.5%)	
Erythrodermic	1 (1.1%)	1 (4.5%)	
Guttate	1 (1.1%)	1 (4.5%)	
Pustular psoriasis	1 (1.1%)	1 (4.5%)	
PASI score	9.56 ± 9.25	14.81 ± 10.17	0.009^1^
PASI score category			0.001^2^
≤12	66 (75.0%)	8 (38.1%)	
>12	22 (25.0%)	13 (61.9%)	
BSA (%)	10.38 ± 13.67	18.27 ± 19.22	0.003^1^
BSA			0.001^2^
≤10%	65 (73.0%)	8 (36.4%)	
>10%	24 (27.0%)	14 (63.6%)	
TSH (µIU/mL)	2.95 ± 1.06	5.83 ± 3.69	0.001^1^
FT3 (pg/dL)	3.05 ± 0.72	2.47 ± 1.16	0.001^1^
FT4 (ng/dL)	1.33 ± 0.36	1.07 ± 0.76	0.007^1^
Anti-TPO (IU/mL)	38.36 ± 23.58	48.23 ± 27.01	0.135^1^
TSH			<0.001^3^
Low	0 (0.0%)	6 (27.3%)	
WNL	89 (100.0%)	0 (0.0%)	
High	0 (0.0%)	16 (72.7%)	
FT3			<0.001^3^
Low	4 (4.5%)	8 (36.4%)	
WNL	84 (94.4%)	13 (59.1%)	
High	1 (1.1%)	1 (4.5%)	
FT4			<0.001^3^
Low	0 (0.0%)	7 (31.8%)	
WNL	87 (97.8%)	13 (59.1%)	
High	2 (2.2%)	2 (9.1%)	
Anti-TPO			0.057^3^
WNL	77 (86.5%)	15 (68.2%)	
High	12 (13.5%)	7 (31.8%)	
TFT			<0.001^3^
WNL	89 (100.0%)	0 (0.0%)	
Subclinical hypothyroidism	0 (0.0%)	7 (31.8%)	
Hypothyroidism	0 (0.0%)	8 (36.4%)	
Subclinical hyperthyroidism	0 (0.0%)	5 (22.7%)	
Hyperthyroidism	0 (0.0%)	2 (9.1%)	

Table 4 presents the comparative clinicodemographic features of normal patients and those with thyroid autoimmunity. Men and patients aged ≤40 years had more autoimmunity than older (>40 years) and female patients (89.5% vs. 10.5% and 73.75% vs. 26.3%, respectively). However, more patients with thyroid autoimmunity showed milder disease in both PASI scores (63.2% vs. 36.8%) and BSA involved (68.4% vs. 31.6%). There was no statistically significant difference between normal patients and those having thyroid autoimmunity regarding gender, age, duration, and severity of psoriasis.

**Table 4:** Association between anti-TPO and clinical parameters. ^1^Wilcoxon-Mann-Whitney U test; ^2^Chi-squared test; ^3^Fisher’s exact test TFT: thyroid function test; PASI: Psoriasis Area and Severity Index; BSA: body surface area; TSH: thyroid-stimulating hormone; WNL: within normal limit; FT3: free T3; FT4: free T4; anti-TPO: anti-thyroid peroxidase

Parameters	Anti-TPO		P-value
	WNL (n = 92)	High (n = 19)	
Age (years)	41.24 ± 14.08	33.21 ± 10.60	0.020^1^
Age			0.071^2^
≤40 years	47 (51.1%)	14 (73.7%)	
>40 years	45 (48.9%)	5 (26.3%)	
Gender			0.233^3^
Male	69 (75.0%)	17 (89.5%)	
Female	23 (25.0%)	2 (10.5%)	
Total duration of illness (years)	5.43 ± 5.67	4.97 ± 4.66	0.835^1^
Total duration of illness			0.721^2^
≤5 years	64 (69.6%)	14 (73.7%)	
>5 years	28 (30.4%)	5 (26.3%)	
Type of psoriasis			1.000^3^
Plaque psoriasis	80 (87.0%)	18 (94.7%)	
Palmoplantar	6 (6.5%)	1 (5.3%)	
Erythrodermic	2 (2.2%)	0 (0.0%)	
Guttate	2 (2.2%)	0 (0.0%)	
Pustular psoriasis	2 (2.2%)	0 (0.0%)	
PASI score	10.67 ± 10.11	10.14 ± 6.96	0.598^1^
PASI score category			0.627^2^
≤12	62 (68.9%)	12 (63.2%)	
>12	28 (31.1%)	7 (36.8%)	
BSA (%)	12.11 ± 16.16	11.16 ± 9.20	0.292^1^
BSA			0.789^2^
≤10%	60 (65.2%)	13 (68.4%)	
>10%	32 (34.8%)	6 (31.6%)	
TSH (µIU/mL)	3.46 ± 2.15	3.82 ± 2.48	0.443^1^
FT3 (pg/dL)	3.04 ± 0.84	2.47 ± 0.79	0.003^1^
FT4 (ng/dL)	1.32 ± 0.48	1.06 ± 0.40	0.020^1^
Anti-TPO (IU/mL)	30.71 ± 12.30	86.84 ± 11.97	<0.001^1^
TSH			0.094^3^
Low	4 (4.3%)	2 (10.5%)	
WNL	77 (83.7%)	12 (63.2%)	
High	11 (12.0%)	5 (26.3%)	
FT3			0.059^3^
Low	7 (7.6%)	5 (26.3%)	
WNL	83 (90.2%)	14 (73.7%)	
High	2 (2.2%)	0 (0.0%)	
FT4			0.656^3^
Low	5 (5.4%)	2 (10.5%)	
WNL	83 (90.2%)	17 (89.5%)	
High	4 (4.3%)	0 (0.0%)	
TFT			0.125^3^
WNL	77 (83.7%)	12 (63.2%)	
Subclinical hypothyroidism	5 (5.4%)	2 (10.5%)	
Hypothyroidism	5 (5.4%)	3 (15.8%)	
Subclinical hyperthyroidism	3 (3.3%)	2 (10.5%)	
Hyperthyroidism	2 (2.2%)	0 (0.0%)	
TFT Impression			0.057^3^
Normal	77 (83.7%)	12 (63.2%)	
Abnormal	15 (16.3%)	7 (36.8%)	

Table 5 highlights the association between the type of psoriasis and thyroid dysfunction. Plaque psoriasis (18) was associated with a higher number of thyroid dysfunction (seven hypothyroidism, five subclinical hypothyroidism, four subclinical hyperthyroidism, and two hyperthyroidism).

**Table 5:** Association between the type of psoriasis and thyroid dysfunction.

Thyroid dysfunction	Type of psoriasis				
	Plaque psoriasis (n = 98)	Palmoplantar (n = 7)	Erythrodermic (n = 2)	Guttate (n = 2)	Pustular psoriasis (n = 2)
Subclinical hypothyroidism	5 (5.1%)	1 (14.3%)	0 (0.0%)	1 (50.0%)	0 (0.0%)
Hypothyroidism	7 (7.1%)	0 (0.0%)	1 (50.0%)	0 (0.0%)	0 (0.0%)
Subclinical hyperthyroidism	4 (4.1%)	0 (0.0%)	0 (0.0%)	0 (0.0%)	1 (50.0%)
Hyperthyroidism	2 (2.0%)	0 (0.0%)	0 (0.0%)	0 (0.0%)	0 (0.0%)

## Discussion

Despite the various updates in the treatment and pathogenesis of psoriasis, the association of thyroid disorders with psoriasis remains obscure. In this study, we investigated the frequency of thyroid dysfunction and its clinical correlation in 111 psoriasis patients. Although no gender predilection has been reported in the literature, in this study, the majority were males (77.5%), with a male-to-female ratio of 3.44:1. This supports other studies where the majority of psoriasis patients were males [5,6]. However, the exclusion of females in most studies might be a reason for this. In this study, the most common age group affected was the third to the fourth decade, and psoriasis was relatively uncommon in the elderly (>60 years). This was consistent with the age distribution of psoriasis reported in previous studies [7,8]. The most common type of psoriasis observed was chronic plaque psoriasis (88.3%). This was in concordance with the study by Bedi et al. and Dogra et al. [9,10]. In this study, the second most common type of psoriasis was palmoplantar psoriasis, which accounted for 6.3% of the total psoriasis cases. This was more than that reported by Bedi et al. (2%). In most studies, palmoplantar psoriasis comprised only 3-4% of all psoriasis cases. In contrast, other studies reported palmoplantar psoriasis to be the most common [11]. Although psoriasis can begin from any site, the most common initial site of involvement observed in this study was the scalp, which is consistent with previous studies reported in the literature [12].

In a recent study, Rana et al. observed deranged thyroid function in 10% of psoriasis patients and reported hypothyroidism and hyperthyroidism in 5.4% and 2.7% of patients, respectively [13]. Similarly, in this study, deranged thyroid function was seen in 19.8% of the patients, of which hypothyroidism was seen in 7.2%, followed by subclinical hypothyroidism (6.3%), subclinical hyperthyroidism (4.5%), hyperthyroidism (1.8%). A greater number of patients with deranged thyroid function showed moderate-to-severe disease compared to euthyroid patients regarding their PASI score as well as BSA involved (51.7% vs. 40.6%; 48.3% vs. 42.5%, respectively), which is consistent with our findings (61.9% vs. 25.0%; 63.6% vs. 27%, respectively). This is in concordance with the case-control study by Arican et al. who found a significantly higher PASI score in psoriasis patients with deranged thyroid function than euthyroid psoriasis patients [14]. They hypothesized that this might be due to the direct or indirect effects of thyroid hormones on the course of psoriasis, and the excessive production of thyroid hormones possibly aggravates psoriasis because of their hyperproliferative effects. In a U.S.-based National Health and Nutrition Examination Survey data (2011-2012) analysis, patients with psoriasis had a risk of increased thyroid function [15]. However, the association was not significant after adjusting confounding variables. Patients with active psoriasis had significantly lower levels of TSH than those without active disease. Using the same data from 2009 to 2014, Liu et al. performed an analysis again, and the odds of having thyroid disease were increased in all adult psoriasis patients [16]. These findings were also in agreement with a nationwide cohort study from Taiwan [17]. However, Gul et al., Robati et al., Arican et al., and Vassilatou et al. did not find any significant difference in thyroid parameters among psoriatic patients [14,18-20].

Du et al. showed near-normal thyroid function among psoriasis vulgaris patients compared to other types of psoriasis. FT3 and FT4 levels were lower in pustular psoriasis and erythrodermic psoriasis patients, respectively [21]. The authors reported that the decrease in FT3 or FT4 in patients with erythrodermic psoriasis was possibly due to diffuse erythema and repeated shedding of scales, causing water and electrolyte disturbances as well as metabolic abnormalities. Hence, such patients can develop the euthyroid sick syndrome, which presents as low T3 or low T4 syndrome and can be easily misdiagnosed as hypothyroidism. On the contrary, in our study, among the various types of psoriasis, plaque-type presented with a more significant number of deranged thyroid hormones, which may be due to more plaque psoriasis than other types of psoriasis. It has been reported that there is an increased prevalence of thyroid dysfunction in palmoplantar pustulosis, but the majority of the patients were smokers, which could interfere with thyroid function [22].

In our study, anti-TPO antibodies were elevated in 17.1% of the patients, with the maximum number of patients having less severe disease. Rana et al. also noted an increase in anti-TPO antibodies in 13.5% of the patients, with most having a milder disease in terms of PASI as well as BSA involved (66.7% vs. 33.3%; 74.4% vs. 25.6%, respectively), which is in agreement with our study [13]. Manvi et al. reported a prevalence of hypothyroidism with elevated anti-TPO in 8.6% of their patients, suggestive of autoimmune thyroid function; however, there was no significant difference in the prevalence of thyroid autoimmunity between psoriatic patients and the normal population [23]. In the Rotterdam Study, an association was not found between anti-TPO positivity and thyroid function with prevalent psoriatic disease [24]. However, they stated that this might be because most participants suffered from a milder disease. However, there was a positive trend between TSH and prevalent psoriatic disease and FT4 and incident psoriatic disease. The authors also conducted a meta-analysis and proposed that an association exists between anti-TPO positivity and AITD with prevalent psoriatic disease [25].

The risk of thyroid dysfunction and autoimmunity increases in those with psoriatic arthritis [26-28]. AITD and the risk of other autoimmune diseases also increase in psoriatic arthritis [24]. This might be explained by the stronger systemic inflammation, which is proved by the higher levels of interleukin-6, CD16+ proinflammatory monocytes, osteoprotegerin, high-sensitive C-reactive protein, and vascular endothelial growth factor in psoriatic arthritis compared to psoriasis alone [29]. The risk is further increased in psoriatic arthritis with polyarticular involvement and longer disease duration.

Alidrisi et al. reported a significantly higher prevalence of ant-TPO antibodies, Tg antibodies, hypoechogenicity, pseudo-nodularity, and increased vascularity in patients with psoriasis compared to the control group, demonstrating a clear association between psoriasis and Hashimoto’s thyroiditis [30]. Psoriasis patients with late-onset (age ≥40 years) and obesity were significantly more likely to have positive TPO antibodies, with a prevalence of 42.1% and 40.7%, respectively. Types of psoriasis, severity, duration, age, gender, smoking status, type 2 diabetes, and personal and family history did not correlate with thyroid autoimmunity. They stated that Hashimoto’s thyroiditis is associated with HLA class II alleles and early-onset psoriasis with class I HLA, specifically the HLA-C allele, which is not implicated in Hashimoto’s susceptibility. In comparison, late-onset psoriasis had no clear HLA association, which may explain the increased prevalence of thyroid antibodies with increasing age. This finding corroborates the study by Rana et al. [13]. They also opined that the higher prevalence of TPO antibodies in obese psoriatic patients might be related to adipokine-derived cytokines, including leptin, which was present in higher concentrations. On the contrary, increased anti-TPO was seen more in patients aged ≤40 years in this current study.

The significance of thyroid dysfunction as a risk factor for the chronicity and severity of psoriasis needs robust evidence. The exact role of thyroid hormones and antibodies in the etiopathogenesis and whether treatment with anti-thyroid drugs can be an option in psoriasis needs confirmation with well-designed experimental and prospective clinical studies demonstrating the effect of these hormones on keratinocytes. Serial measurements of thyroid hormones and antibodies are required to further characterize the relationship. The geographical and genetic link of these two diseases and the other associated factors such as diabetes, obesity, and stress that can affect thyroid function needs to be studied.

Limitations

Because this was an observational, cross-sectional study, the temporal relationship between thyroid diseases and psoriasis could not be established. Data at a single time point could not reflect long-term exposure to various biochemical factors that might have been important effect modifiers of thyroid disease. Our study did not include a control group of healthy individuals and there were no psoriatic arthritis patients in the study. Additionally, serial thyroid measurement and ultrasonography were not done.

## Conclusions

This study aimed to expand our knowledge of psoriasis and its association with thyroid dysfunction, thyroid autoimmunity, and clinical features, including age, gender, disease duration, and severity of psoriasis. The present study illustrated deranged thyroid status and increased anti-TPO in 19.8% and 17.1% of psoriasis patients, respectively, suggesting thyroid dysfunction and autoimmunity. Dermatology, endocrinology, and rheumatology consultations are warranted for the early identification of thyroid diseases in psoriasis and psoriatic arthritis patients to provide better clinical care and management.

## References

[1] Wu JJ, Nguyen TU, Poon KY, Herrinton LJ (2012). The association of psoriasis with autoimmune diseases. J Am Acad Dermatol.

[2] Hoath SB, Lakshmanan J, Scott SM, Fisher DA (1983). Effect of thyroid hormones on epidermal growth factor concentration in neonatal mouse skin. Endocrinology.

[3] Safer JD, Fraser LM, Ray S, Holick MF (2001). Topical triiodothyronine stimulates epidermal proliferation, dermal thickening, and hair growth in mice and rats. Thyroid.

[4] Ruffilli I, Ragusa F, Benvenga S, Vita R, Antonelli A, Fallahi P, Ferrari SM (2017). Psoriasis, psoriatic arthritis, and thyroid autoimmunity. Front Endocrinol (Lausanne).

[5] Bedi TR (1977). Psoriasis in North India. Geographical variations. Dermatologica.

[6] Kaur I, Kumar B, Sharma KV, Kaur S (1986). Epidemiology of psoriasis in a clinic from North India. Indian J Dermatol Venereol Leprol.

[7] Lal S (1966). Clinical pattern of psoriasis in Punjab. Indian J Dermatol Venereol.

[8] Mehta TK, Shah RN, Marquis L (1976). A study of 300 cases of psoriasis. Indian J Dermatol Venereol Leprol.

[9] Dogra S, Yadav S (2010). Psoriasis in India: prevalence and pattern. Indian J Dermatol Venereol Leprol.

[10] Bedi TR (1995). Clinical profile of psoriasis in North India. Indian J Dermatol Venereol Leprol.

[11] Venkatesan A, Aravamudhan R, Perumal SK, Kannan R, Thirunavukkarasu V, Shukla S (2015). Palmoplantar psoriasis- ahead in the race-a prospective study from a tertiary health care centre in South India. J Clin Diagn Res.

[12] Kaur I, Handa S, Kumar B (1997). Natural history of psoriasis: a study from the Indian subcontinent. J Dermatol.

[13] Rana A, Mahajan VK, Chauhan PS, Mehta KS, Sharma SB, Sharma A, Sharma R (2020). The association of thyroid dysfunction with chronic plaque psoriasis: a hospital-based retrospective descriptive observational study. Indian Dermatol Online J.

[14] Arican O, Bilgic K, Koc K (2004). The effect of thyroid hormones in psoriasis vulgaris. Indian J Dermatol Venereol Leprol.

[15] Lai YC, Yew YW (2016). Psoriasis and thyroid profile: Analysis of the U.S. National Health and Nutrition Examination Survey database. Indian J Dermatol Venereol Leprol.

[16] Liu J, Thatiparthi A, Martin A, Wu JJ (2021). Association between psoriasis and thyroid dysfunction among US adults in the 2009-2014 National Health and Nutrition Examination Survey. J Am Acad Dermatol.

[17] Wang SH, Wang J, Lin YS, Tung TH, Chi CC (2019). Increased risk for incident thyroid diseases in people with psoriatic disease: a cohort study. J Am Acad Dermatol.

[18] Gul U, Gonul M, Kaya I, Aslan E (2009). Autoimmune thyroid disorders in patients with psoriasis. Eur J Dermatol.

[19] Robati RM, Toossi P, Rahmati-Roodsari M, Khalilazar S, Abolhasani E, Namazi N, Younespour S (2013). Association of psoriasis severity with serum prolactin, thyroid hormones, and cortisol before and after treatment. ScientificWorldJournal.

[20] Vassilatou E, Papadavid E, Papastamatakis P (2017). No association of psoriasis with autoimmune thyroiditis. J Eur Acad Dermatol Venereol.

[21] Du J, Ma C, Wang R, Lin L, Gao L, Chen S, Lu X (2021). Relationship between different psoriasis types and thyroid dysfunction: a retrospective analysis. Scanning.

[22] Giménez-García R, Sánchez-Ramón S, Cuellar-Olmedo LA (2003). Palmoplantar pustulosis: a clinicoepidemiological study. The relationship between tobacco use and thyroid function. J Eur Acad Dermatol Venereol.

[23] Manvi S, Mahajan VK, Mehta KS, Yadav RS, Bhushan S, Chauhan PS (2019). Psoriasis and co-morbidities: is hyperhomocystienemia the common link?. J Assoc Physicians India.

[24] Khan SR, Bano A, Wakkee M (2017). The association of autoimmune thyroid disease (AITD) with psoriatic disease: a prospective cohort study, systematic review and meta-analysis. Eur J Endocrinol.

[25] Antonelli A, Delle Sedie A, Fallahi P (2006). High prevalence of thyroid autoimmunity and hypothyroidism in patients with psoriatic arthritis. J Rheumatol.

[26] Kiguradze T, Bruins FM, Guido N (2017). Evidence for the association of Hashimoto's thyroiditis with psoriasis: a cross-sectional retrospective study. Int J Dermatol.

[27] Tsai TF, Wang TS, Hung ST, Tsai PI, Schenkel B, Zhang M, Tang CH (2011). Epidemiology and comorbidities of psoriasis patients in a national database in Taiwan. J Dermatol Sci.

[28] Fallahi P, Ferrari SM, Ruffilli I (2017). Increased incidence of autoimmune thyroid disorders in patients with psoriatic arthritis: a longitudinal follow-up study. Immunol Res.

[29] Paek SY, Han L, Weiland M (2015). Emerging biomarkers in psoriatic arthritis. IUBMB Life.

[30] Alidrisi HA, Al Hamdi K, Mansour AA (2019). Is there any association between psoriasis and Hashimoto's thyroiditis?. Cureus.

